# Primary Retrovesical Leiomyosarcoma: A Case Report

**DOI:** 10.7759/cureus.69422

**Published:** 2024-09-14

**Authors:** Raquel Rudy, David I LeRoy, Jared Goldberg

**Affiliations:** 1 Internal Medicine, Ascension Macomb-Oakland Hospital, Warren, USA

**Keywords:** acute gastrointestinal bleed, pelvic mass, pelvic retroperitoneal leiomyosarcoma, soft tissue sarcoma, warburg effect

## Abstract

Soft tissue sarcomas (STS) are a rare group of malignant tumors in adults. This group of tumors contains a variety of subtypes, each with distinct clinical features and presentations. Leiomyosarcoma is among the most common subtypes, which typically occur within the uterus, retroperitoneum, abdomen, and large blood vessels. At presentation, tumors are often large and may have metastasized to the lungs, liver, or peritoneum. Given the rarity and variability of the disease, a multidisciplinary treatment approach is essential for management, however, further research is needed to develop histologic-specific guidelines and targeted treatments.

Here, we present a case of a 69-year-old male who was found to have a large pelvic mass of unknown etiology after presenting to the emergency department with generalized weakness, decreased appetite, and an inability to ambulate. On examination, he was found to have testicular swelling and trace edema of bilateral lower extremities. Labs significant for lactic acidosis and CT chest/abdomen/pelvis showed a large heterogeneously enhancing mass within the pelvis measuring up to 15 cm x 12 cm x 15 cm, of uncertain origin, but highly concerning for malignancy. Biopsy with immunohistochemical stains of the mass revealed a grade 2 leiomyosarcoma. Due to the size and location of the mass, transfer to a tertiary care center was recommended. Computed tomography angiography (CTA) abdomen/pelvis and magnetic resonance imaging (MRI) abdomen/pelvis performed at the tertiary care hospital revealed a 16.6 cm heterogeneously enhancing, necrotic mass within the retrovesical pelvic space and three liver lesions, which were concerning for metastases. Due to the patient’s deconditioning and poor functional status, surgical resection and radiation were not offered. The patient expired soon after his code status was changed to comfort care.

## Introduction

Soft tissue sarcomas (STS) are a rare group of malignant tumors that account for less than 1% of all adult malignancies [1,2]. This group of tumors contains nearly 100 histologic subtypes, each with distinct clinical features and presentations [3]. Evaluation for suspected STS involves a thorough history, physical exam, imaging, and often, biopsy of the mass. Leiomyosarcoma is among the most common subtypes, and it comprises up to 10%-20% of newly diagnosed STS [4]. Leiomyosarcomas most commonly arise in the uterus, retroperitoneum, abdomen, and larger blood vessels [5]. Sarcomas originating within the retrovesical space are extremely rare [6]. These tumors typically metastasize to the lung; however, the primary tumors within the abdominal cavity more commonly metastasize to the liver and peritoneum [2]. The location of the disease is an important factor in the determination of the appropriate treatment regimen and outcome of the disease [2]. Given the rarity and variability of disease, a multidisciplinary treatment approach is essential for the management of these patients. Here, we detail a case of a retrovesical leiomyosarcoma with liver metastases in a 69-year-old male and discuss the difficulties of diagnosis and management in the setting of STS.

## Case presentation

**Figure 1:**
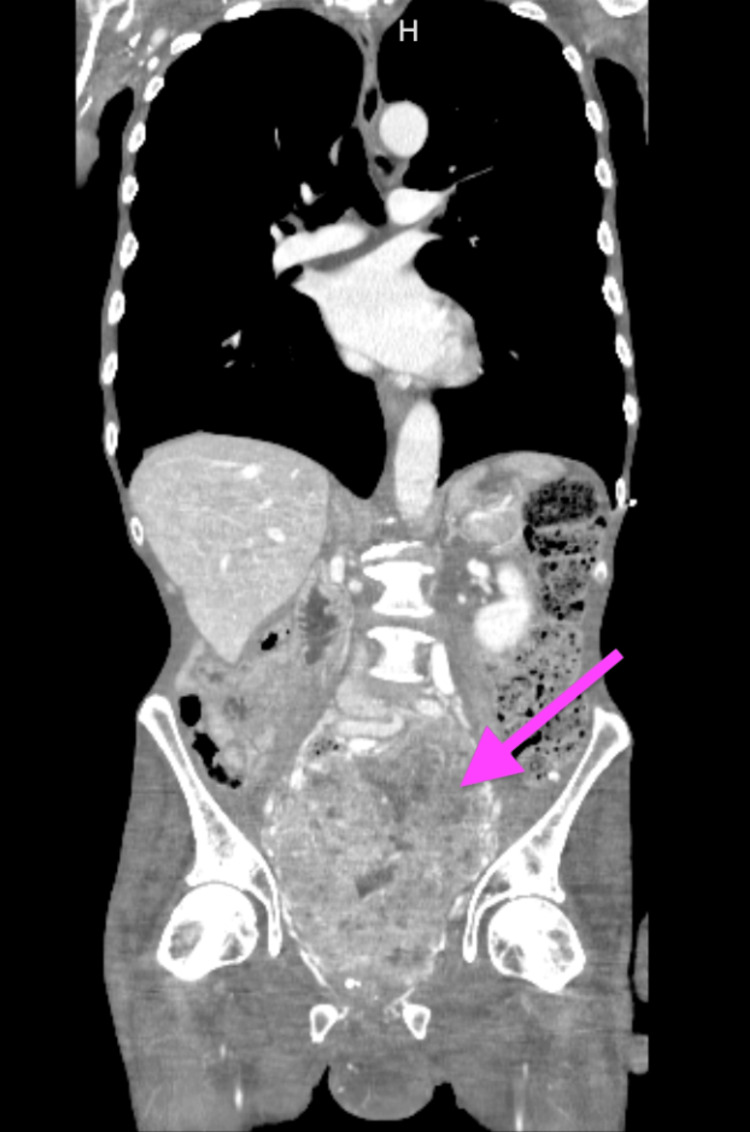
CT chest/abdomen/pelvis demonstrates 15 cm x 12 cm x 15 cm heterogeneously enhancing pelvic mass

**Figure 2:**
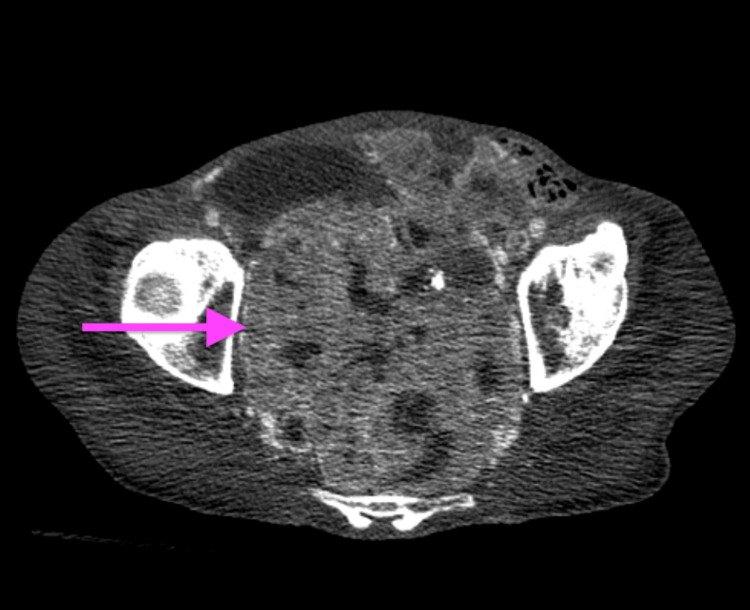
CT chest/abdomen/pelvis axial view of 15 cm x 12 cm x 15 cm heterogeneously enhancing pelvic mass

A 69-year-old male with a medical history significant for hypertension, hyperlipidemia, alcohol use disorder, and a history of cerebrovascular accident presented to the emergency department with generalized weakness, decreased appetite, stool incontinence, and an inability to ambulate for several days. On examination, he was found to have testicular swelling with induration, a palpable pelvic mass, and trace edema of bilateral lower extremities. Labs were significant for lactic acidosis of 10.1 on arrival. CT chest/abdomen/pelvis with contrast showed a large heterogeneously enhancing lobulated mass within the pelvis measuring up to 15 cm x 12 cm x 15 cm, of uncertain origin, but highly concerning for malignancy (Figures 1, 2). Imaging was negative for additional metastatic or primary malignancy in the chest, abdomen, or pelvis.

On recommendation by oncology, tumor markers including AFP, CEA, Beta-hCG, and PSA were measured and were all within normal. During the hospital course, lactic acidosis continued to worsen despite aggressive fluid resuscitation. Causes of lactic acidosis were investigated and ruled out including hypoperfusion, infection, and adverse medication reactions. Therefore, it was believed that the lactic acidosis was primarily due to the “Warburg” effect from the pelvic mass. The final lactic acid level prior to transfer to the tertiary hospital was 20.0.

**Figure 3:**
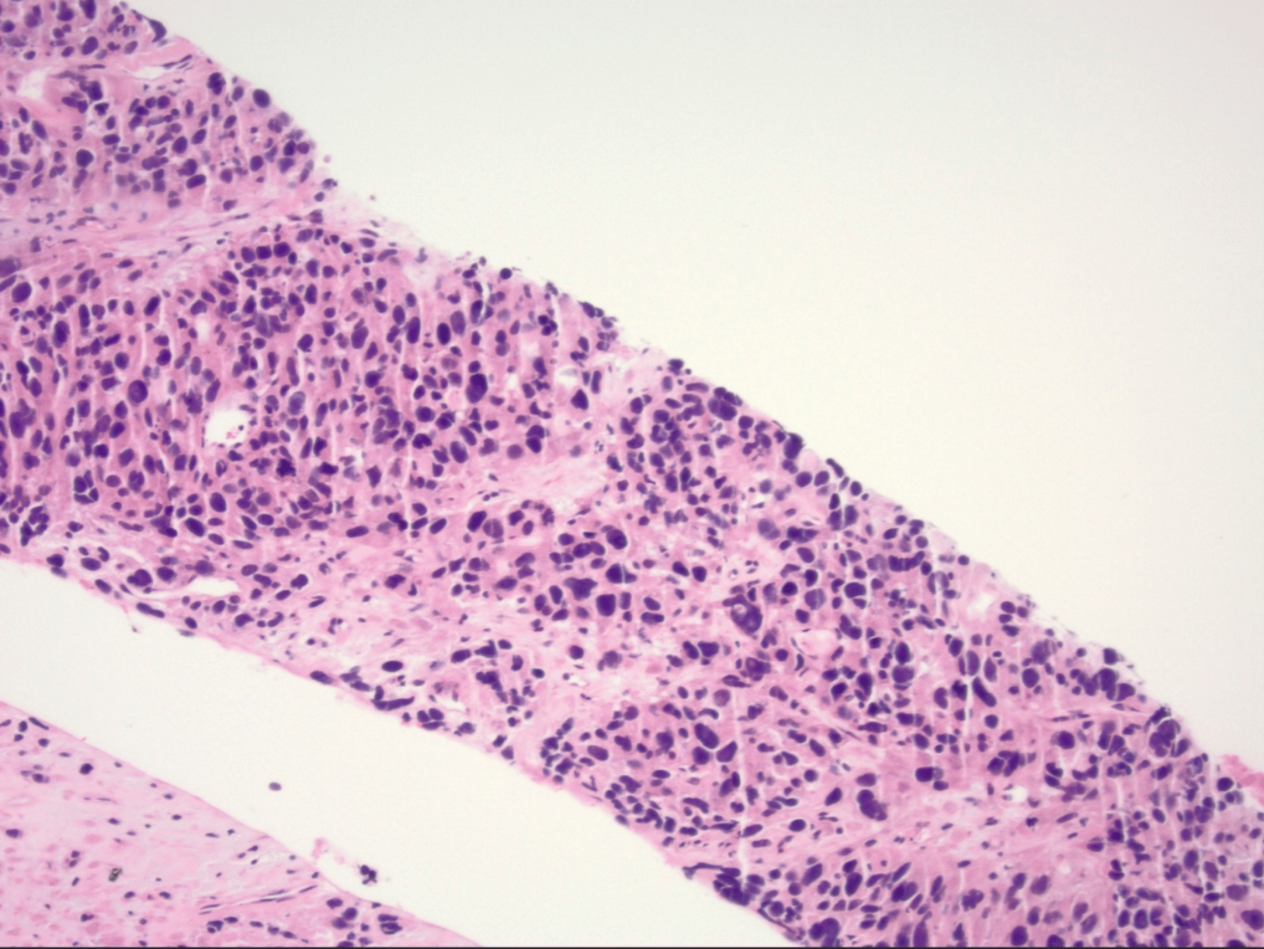
Low magnification of the tissue, which shows a cellular neoplasm composed of stromal cells with marked nuclear pleomorphism and moderate cytoplasm. No epithelial differentiation such as squamous or glandular is seen

**Figure 4:**
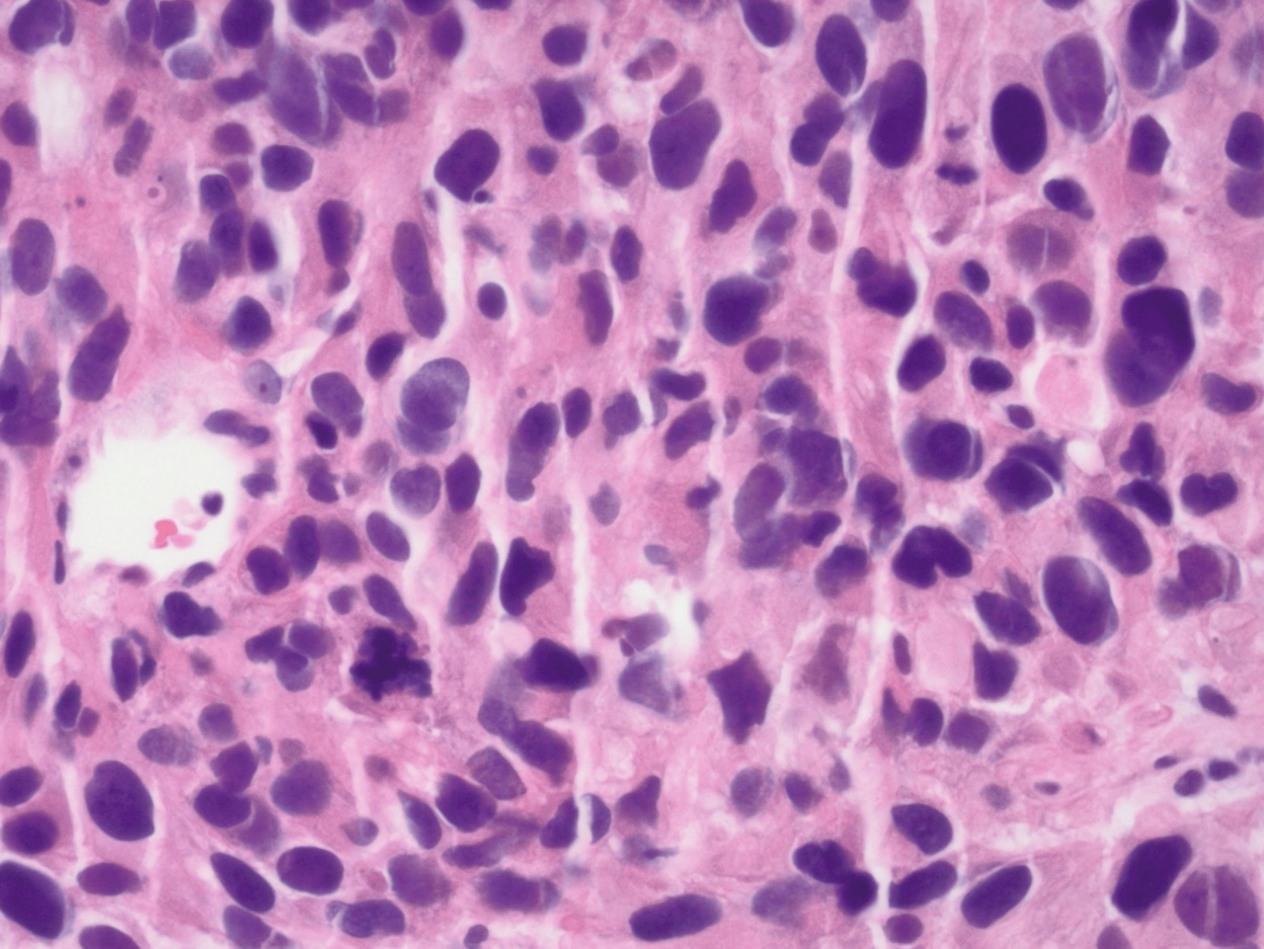
Higher magnification of tissue highlights the marked nuclear atypia and an abnormal mitotic figure

**Figure 5:**
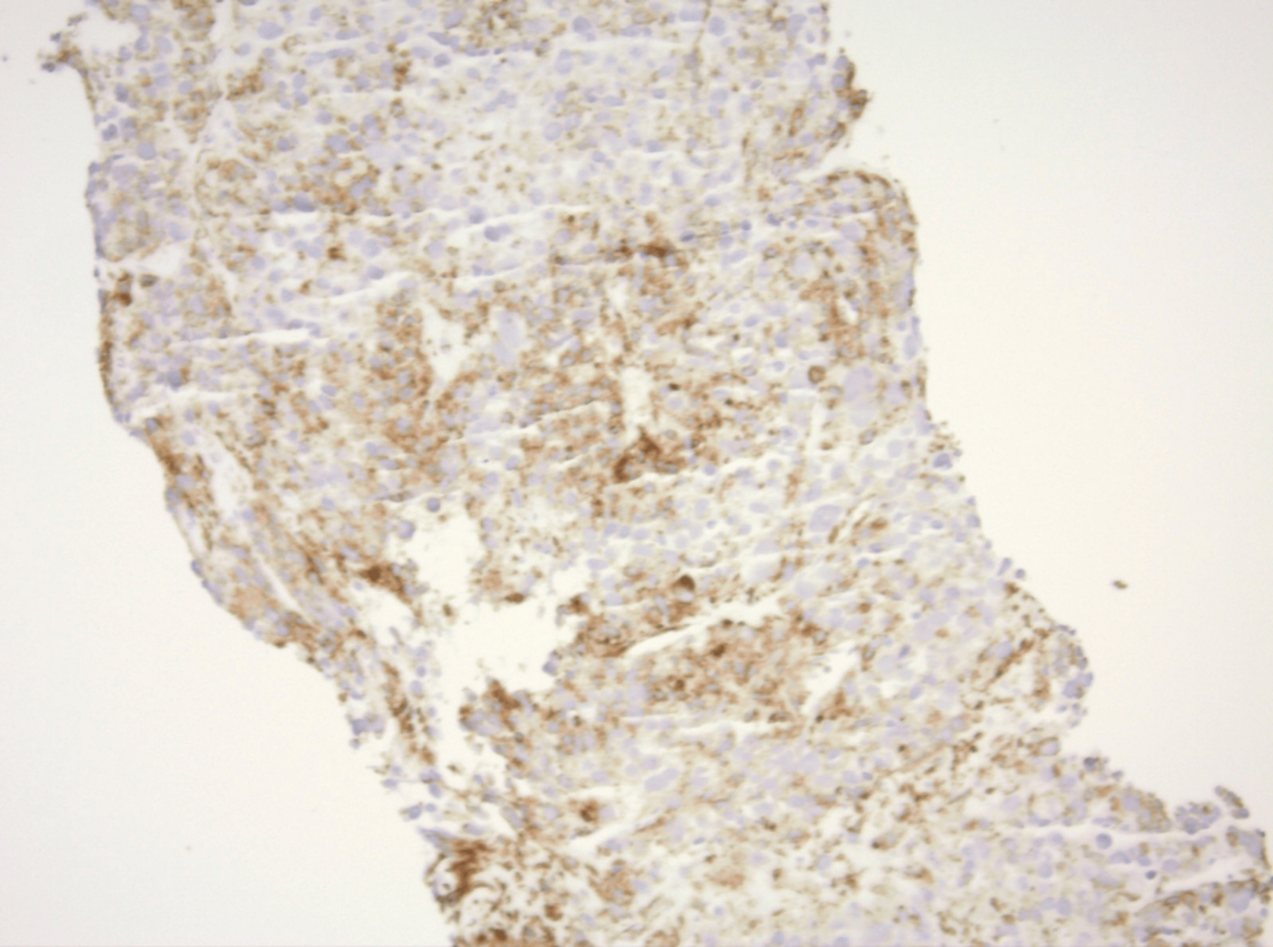
Immunohistochemical staining of the tissue shows brown staining of the tumor with smooth muscle actin, supporting a diagnosis of a tumor of smooth muscle origin

A biopsy of the pelvic mass performed by interventional radiology revealed grade 2 leiomyosarcoma (Figures 3, 4, 5). Immunochemical stains were positive for smooth muscle actin, HHF35, and desmin and negative for pankeratin, arginase, CD117, CD30, placental alkaline phosphatase (PLAP), myoglobin, and SOX10. The general surgery service was consulted for evaluation of tumor resection, but due to the size and location of the mass, they recommended transfer to a tertiary care center for resection given the complexity of the surgery that would be required to remove the mass. 

Once he was transferred to the tertiary care hospital, his hospital course was complicated by acute thrombosis of the left peroneal, right axillary, and right brachial deep vein. The patient was started on anticoagulation with intravenous heparin infusion. However, shortly after, he developed acute blood loss anemia with a drop in hemoglobin from 9.0 gm/dL to 6.4 gm/dL within 48 hours of initiating heparin. One unit of packed red blood cells was transfused and heparin was discontinued. Computed tomography angiography (CTA) of the abdomen and pelvis was performed to locate a source of bleeding, but none was found. However, in addition to re-demonstrating the pelvis mass, the CTA identified two low-attenuation lesions in the liver. Magnetic resonance imaging (MRI) of the abdomen and pelvis confirmed the large necrotic mass within the retrovesical pelvic space measuring 16.6 cm as well as three liver lesions, concerning metastatic disease. Due to the patient’s deteriorating functional status and deconditioning, surgical oncology was unable to offer surgical resection. Radiation oncology recommended inpatient physical rehabilitation before radiation would be offered. However, the patient continued to deteriorate and began having numerous complications including hypoxia and persistent hypoglycemia, which was unable to be corrected despite various interventions. The patient met with palliative care and elected to proceed with only comfort measures and died shortly after.

## Discussion

This case exemplifies the importance of accurate diagnoses in the management of complex or rare malignancies as well as the importance of multidisciplinary involvement among many specialist physicians. An accurate diagnosis is crucial for prognosis and management. Unfortunately, diagnosis of soft tissue tumors presents a challenge due to their rarity and heterogeneity. In fact, between 10%-30% of sarcomas are inaccurately diagnosed, which may lead to inappropriate management [7,8]. Initial evaluation for suspected STS involves a thorough history, physical exam, and imaging. Prior to treatment, a biopsy for confirmation of histologic subtype and grade must be completed, along with immunohistochemical studies, cytogenetics, and molecular testing [2,9]. Immunohistochemical staining positive for at least two of the following markers is suggestive of a diagnosis of leiomyosarcoma, which includes desmin, smooth muscle actin, muscle actin HHF-35, h-caldesmon, smooth muscle myosin, or calponin [10]. 

Multidisciplinary management, in particular at high-volume centers or specialized sarcoma centers, is essential for reducing mortality and improving overall survival in these patients with STS [11]. Multidisciplinary teams often include radiologists, pathologists, radiation, surgical, and medical oncologists in order to ensure correct diagnosis, and collaboration for treatment planning. Surgical resection remains the treatment of choice in localized disease with neoadjuvant radiation preferred in select cases [2]. Surgical resections with wide margins are the preferred method; however, given the large size and location in the retroperitoneum, as in our patient’s case, resection is often difficult [5]. Due to the close proximity to other structures in the retroperitoneum, complete surgical resection is achieved in less than 70% of patients [2]. Patients who underwent complete resection with wide margins have a median survival of 103 months, compared to 18 months in those who underwent incomplete resection [12]. For patients with advanced, unresectable, or metastatic disease, single-agent chemotherapy or anthracycline-based combination chemotherapy with doxorubicin or epirubicin with ifosfamide and/or dacarbazine is most commonly used [2]. Progression-free survival and overall survival with the use of chemotherapy is dependent on the combination of chemotherapeutic agents used but is significantly less than that of those who underwent surgical excision [2]. In more recent years, targeted therapies, such as pazopanib, a tyrosine-kinase inhibitor, have shown promise in certain types of advanced or metastatic disease who failed at least one anthracycline-based chemotherapy regimen [2,13]. 

## Conclusions

STS are a rare and complex group of tumors that account for less than 1% of all adult malignancies. The heterogeneity and overall rarity of these tumors often present challenges to ensure correct diagnosis and management in these patients. Therefore, multidisciplinary care at a high-volume or specialized sarcoma center should be sought out at the time of diagnosis to reduce mortality and improve prognosis. An updated classification of soft tissue tumors based on histology remains essential for diagnostic accuracy. Although management has shifted toward histology-specific treatment strategies recently, the need for collaborative research remains to develop treatment guidelines for each subtype and develop targeted therapies. 
